# CIRPMC: An online model with simplified inflammatory signature to predict the occurrence of critical illness in patients with COVID‐19

**DOI:** 10.1002/ctm2.210

**Published:** 2020-10-21

**Authors:** Yue Gao, Lingxi Chen, Shaoqing Zeng, Xikang Feng, JianHua Chi, Ya Wang, Huayi Li, Tengping Jiang, Yang Yu, XiaoFei Jiao, Dan Liu, XinXia Feng, SiYuan Wang, RuiDi Yu, Yuan Yuan, Sen Xu, Guangyao Cai, Xiaoming Xiong, Pingbo Chen, Qingqing Mo, Xin Jin, Yuan Wu, Ding Ma, Chunrui Li, Shuai Cheng Li, Qinglei Gao

**Affiliations:** ^1^ National Medical Center for Major Public Health Events Tongji Hospital Tongji Medical College Huazhong University of Science and Technology Wuhan People's Republic of China; ^2^ Cancer Biology Research Center (Key Laboratory of Chinese Ministry of Education) Tongji Hospital Tongji Medical College Huazhong University of Science and Technology Wuhan People's Republic of China; ^3^ Department of Gynecology and Obstetrics Tongji Hospital Tongji Medical College Huazhong University of Science and Technology Wuhan People's Republic of China; ^4^ Department of Computer Science City University of Hong Kong Kowloon Tong Hong Kong; ^5^ School of Software Northwestern Polytechnical University Xi'an People's Republic of China; ^6^ State Key Laboratory of Information Engineering in Surveying Mapping and Remote Sensing (LIESMARS) Wuhan University Wuhan China; ^7^ Department of Gastroenterology Tongji Hospital Tongji Medical College Huazhong University of Science and Technology Wuhan People's Republic of China; ^8^ Department of Hematology Tongji Hospital Tongji Medical College Hua Zhong University of Science and Technology Wuhan China; ^9^ Department of Biomedical Engineering City University of Hong Kong Kowloon Tong Hong Kong

Dear editor,

The coronavirus disease 2019 (COVID‐19) is characterized by heterogeneous clinical features and multiple organ damage. Many patients with mild symptoms can suddenly develop into critical illness and progress to a refractory state that has significantly increased mortality, indicating the necessity to promptly identify patients at high risk of physiologic deterioration before the occurrence of critical COVID‐19. With both innate and adaptive immune compartments contribution, cytokine storm in covid‐19 is widely concerned. Hyperinflammatory response induced by immune dysfunction is reported to underpin critical COVID‐19.^1^ Uncontrolled release of cytokines results in tissue damage and further leads to multiple organ failure, which is the major cause of death in patients with COVID‐19.^2^ As expected, the differences of multiple cytokines and immune features between critical ill and noncritical ill patients were observed in clinical practice. Besides, early seroconversion and high antibody titer were linked with less severe clinical symptoms. Using inflammatory/immune factors to predict the risk of developing critical COVID‐19 under the assistance of machine learning (ML) is promising to aid management of the disease, but rarely reported.

Electronic health records (EHRs) harbor valuable resources generated from routine medical activities and have been widely used. However, medical data are often complex, multidimensional, nonlinear, heterogeneous, and required to be analyzed using more effective statistical methods than traditional logistic regression. ML is a subfield of artificial intelligence that encapsulates statistical and mathematical algorithms, which enables facts interrogation and complex decision making through a given set of data. The combination of EHRs and ML shows potential applications in predicting the risk of atherosclerotic cardiovascular disease and gestational diabetes.

In this multicenter study, we developed an online model with four inflammatory factors (C reactive protein [CRP], tumor necrosis factor α [TNF‐α], interleukin 2 receptor [IL‐2R], and interleukin 6 [IL‐6]) that enabled accurate identification of COVID‐19 patients prone to critical illness approximately 20 days in advance. The model was validated in an internal validation cohort (SFV cohort) and an external validation cohort (OV cohort). Study design is presented in Figure 2A. The detailed demographic and characteristics of patients are shown in Table S1.

FIGURE 1Feature selection by LASSO and model performance across cohorts. A, LASSO variable trace profiles of the eight features. The vertical dashed line shows the best lambda value (0.035) chosen by 10‐fold cross validation. B, Feature with zero coefficient (colored with gray) at lambda = 0.035 was considered less crucial to the patient's critical illness status and removed by Lasso logistic regression analysis. Feature with positive coefficient (colored with red) is regarded high risk in respect to critical illness. C, D, ROC curve and AUC of SVM, LR, GBDT, KNN, and NN in SFV cohort and OV cohort, respectively. E, F, KM curve of low‐risk and high‐risk subgroups predicted by SVM model in SFV and OV cohorts, respectively. The light red or blue areas refer to the 95% confidence interval. *P* value is computed by log‐rank test. Hazard ratio (HR) and its 95% confidence interval are obtained with univariate Cox model. Abbreviations: CRP, C reactive protein; GBDT, gradient boosted decision tree; HR, hazard ratio; IL‐2R, interleukin 2 receptor; IL‐6, interleukin 6; IL‐8, interleukin 8; IL‐10, interleukin 10; IL‐1β, interleukin 1β; KNN, k‐nearest neighbor; LASSO, least absolute shrinkage and selection operator; LR, logistic regression; NN, neural network; OV cohort, external validation cohort of Optical Valley Campus of Tongji Hospital; SFV cohort, internal validation cohort of Sino‐French New City Campus of Tongji Hospital; SVM, supported vector machine; TNF‐α, tumor necrosis factor α

**Figure:**
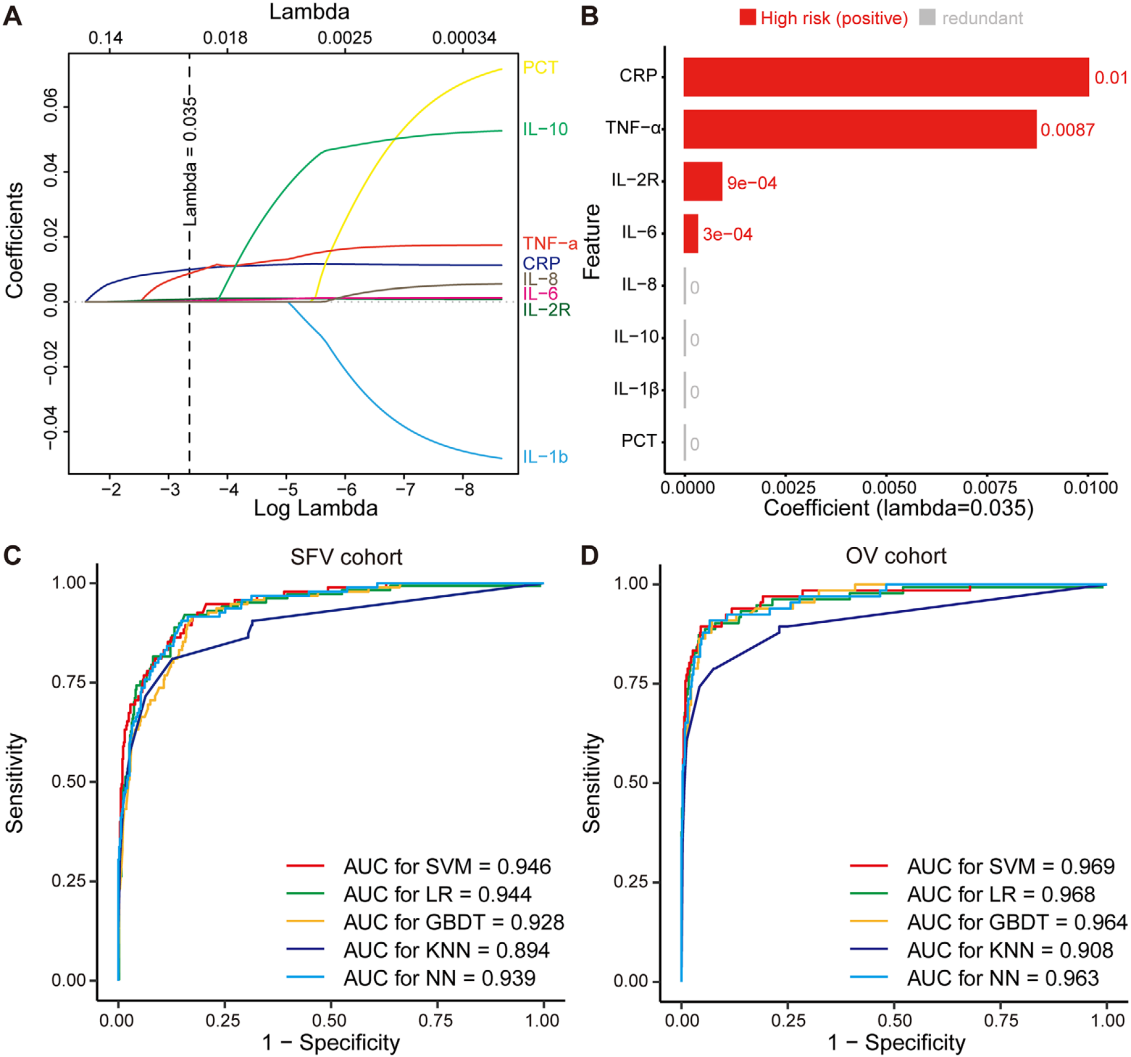

**Figure:**
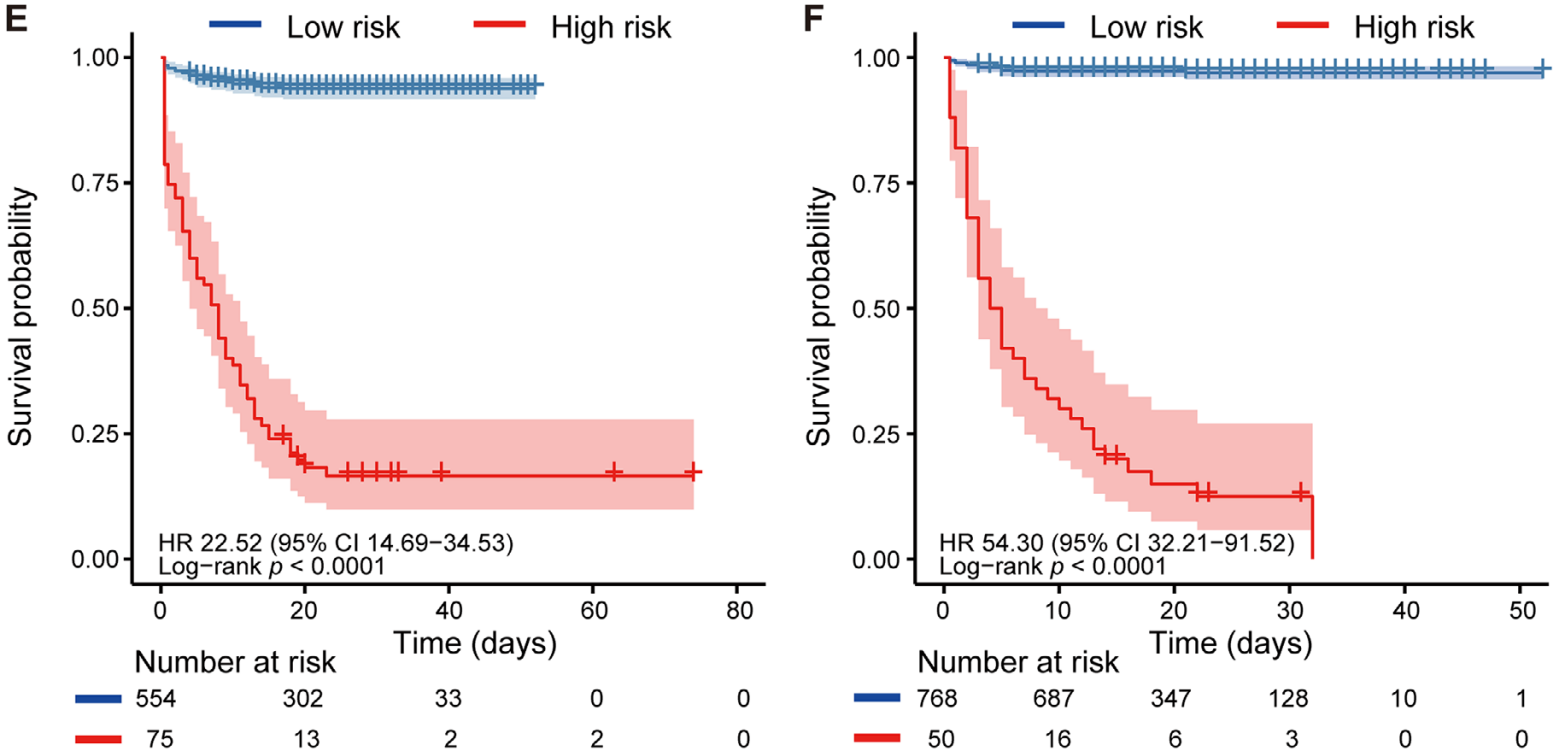

A total of 15 raw inflammatory/immune features were collected from COVID‐19 patients at admission. After feature filtering (Figure S1) and data imputation (Figure S2),^3^ eight features were fitted into Least Absolute Shrinkage and Selection Operator (LASSO)^4^ logistic regression for feature selection (Figure 1A). As illustrated in Figure 1B, we considered features whose coefficients equaled to zero as redundant and less predictive features. As a result, LASSO analysis identified four features (CRP, TNF‐α, IL‐2R, and IL‐6) for the development of critical illness classifier.

We conducted the Spearman correlation analysis between the four features and critical illness status. Figure S3A indicates that the positive correlation at varying degrees existed across five features. The top weighted features, IL‐6 (*R* = 0.49), CRP (*R* = 0.47), IL‐2R (*R* = 0.43), and TNF‐α (*R* = 0.37), were consisted with previously reported risk factors that were highly correlated with poor outcome of COVID‐19. Standard box plots presented significant differences (*P* < 2.2e‐16) of the four features between critically ill and noncritically ill COVID‐19 patients (Figure S3B). The median (IQR) expression of TNF‐α (17.0, 10.5‐29.3, pg/mL), CRP (182.7, 103.2‐258.6, mg/L), IL‐2R (1447.0, 993.0‐2327.5, U/mL), and IL‐6 (169.4, 58.1‐640.9, pg/mL) was significantly higher in critically ill patients compared with TNF‐α (8.1, 6.1‐10.5, pg/mL), CRP (16.1, 2.5‐57.8, mg/L), IL‐2R (520.0, 299.0‐770.5, U/mL), and IL‐6 (5.0, 2.1‐18.4, pg/mL) in noncritically ill patients.

During model development stage, five models (support vector machine [SVM], logistic regression [LR], gradient boosted decision tree [GBDT], k‐nearest neighbor [KNN], and neural network [NN]) were trained for risk prediction. In general, all five models showed varying but promising critical illness risk prediction performance in the internal and external validation cohorts. CIRPMC (critical illness risk prediction model for COVID‐19) derived from SVM achieved the highest predictive performance. Relative feature importance rank of SVM is shown in Figure S4. As binary classifier, CIRPMC outputted critical illness risk probability (*P*) ranged from 0 to 1 for each patient, and stratified patients with *P* < .5 as low risk, otherwise high risk. For SFV cohort, CIRPMC achieved an AUC (area under the receiver operating characteristics curve) of 0.946 (95% CI 0.923‐0.969) to identify patients having high risk of developing critical illness with an accuracy of 92.7% (95% CI 90.4%‐94.6%). For OV cohort, CIRPMC demonstrated an AUC of 0.969 (95% CI 0.945‐0.992) and an accuracy of 96.6% (95% CI 95.1‐97.7%) (Figure 1C, D). The calibration curve of CIRPMC in two validation cohorts is depicted in Figure S5. Intriguingly, CIRPMC also displayed the minimal Brier score of 0.057 for SFV cohort and 0.028 for OV cohort. All other metrics and the performance of other models are listed in Table 1.

**TABLE 1:** Performance metrics for critical illness risk prediction of models in cohorts

	AUC (95% CI)	Accuracy (95% CI)	SN (95% CI)	SP (95% CI)	PPV (95% CI)	NPV (95% CI)	Kappa	F1	Brier
SFV cohort									
SVM	0.946 (0.923‐0.969)	0.927 (0.904‐0.946)	0.653 (0.548‐0.747)	0.976 (0.959‐0.987)	0.827 (0.722‐0.904)	0.940 (0.917‐0.959)	0.688	0.729	0.057
LR	0.944 (0.921‐0.968)	0.916 (0.891‐0.936)	0.758 (0.659‐0.840)	0.944 (0.921‐0.962)	0.706 (0.608‐0.792)	0.956 (0.935‐0.972)	0.681	0.731	0.067
GBDT	0.928 (0.902‐0.954)	0.900 (0.874‐0.922)	0.663 (0.559‐0.757)	0.942 (0.919‐0.960)	0.670 (0.566‐0.764)	0.940 (0.917‐0.959)	0.608	0.667	0.077
KNN	0.894 (0.853‐0.934)	0.913 (0.888‐0.933)	0.579 (0.473‐0.680)	0.972 (0.954‐0.984)	0.786 (0.671‐0.875)	0.928 (0.904‐0.948)	0.618	0.667	0.068
NN	0.939 (0.915‐0.963)	0.849 (0.819‐0.876)	0.000 (0.000‐0.038)	1.000 (0.993‐1.000)	NA (0.000‐1.000)	0.849 (0.819‐0.876)	0.000	NA	0.085
OV cohort									
SVM	0.969 (0.945‐0.992)	0.966 (0.951‐0.977)	0.667 (0.540‐0.778)	0.992 (0.983‐0.997)	0.880 (0.757‐0.955)	0.971 (0.957‐0.982)	0.741	0.759	0.028
LR	0.968 (0.946‐0.989)	0.963 (0.948‐0.975)	0.803 (0.687‐0.891)	0.977 (0.964‐0.987)	0.757 (0.640‐0.852)	0.983 (0.971‐0.991)	0.759	0.779	0.034
GBDT	0.964 (0.944‐0.9845)	0.958 (0.942‐0.971)	0.773 (0.653‐0.870)	0.975 (0.961‐0.985)	0.729 (0.609‐0.828)	0.980 (0.967‐0.989)	0.727	0.750	0.041
KNN	0.908 (0.861‐0.954)	0.957 (0.941‐0.970)	0.606 (0.478‐0.724)	0.988 (0.977‐0.995)	0.816 (0.680‐0.912)	0.966 (0.951‐0.978)	0.673	0.696	0.037
NN	0.963 (0.940‐0.986)	0.919 (0.899‐0.937)	0.000 (0.000‐0.054)	1.000 (0.995 – 1.000)	NA (0.000‐1.000)	0.919 (0.899‐0.937)	0.000	NA	0.052

Abbreviations: AUC, area under the curve; SVM, supported vector machin; GBDT, gradient boosted decision tree; KNN, k‐nearest neighbour; LR, logistic regression; NN, neural network; NPV, negative predictive value; OV, Optical Valley Campus of Tongji Hospital; PPV, positive predictive value; SFV cohort, internal validation cohorts of Sino‐French New City Campus of Tongji Hospital; SN, sensitivity; SP, specificity.

With critical illness as status and time from admission to critical illness or discharge as the endpoint, Kaplan‐Meier analysis further confirmed the risk stratification ability of the model. CIRPMC robustly stratified high‐risk patients and low‐risk patients with *P* < .0001 in both internal and external validation cohorts. The univariate Cox analysis also demonstrated the positive correlation between CIRPMC predicted critical illness subgroup and the ground truth critical illness survival for internal (HR: 22.52, 95% CI 14.69‐34.53) and external (HR:54.30, 95% CI 32.21‐91.52) validation cohorts, respectively (Figure 1E, F).

Additionally, we opened up an online calculator based on CIRPMC to input the values of features needed for risk prediction of COVID‐19 patients (https://cirpmc.deepomics.org/). After the clinicians fill in the online form with corresponding features, CIRPMC returns a personalized probability and risk group of critical illness. Illustration of an example of the online prediction system is presented in Figure 2B. In this study, CIRPMC was developed to identify COVID‐19 patients with high risk of developing critical illness and achieved high predictive performance with an AUC range from 0.946 to 0.969 across the internal and external validation cohorts. The accurate and rapid risk stratification is critical to ensure health systems agile and hopefully will optimize patient outcomes where “time is life.”

**FIGURE 2:**
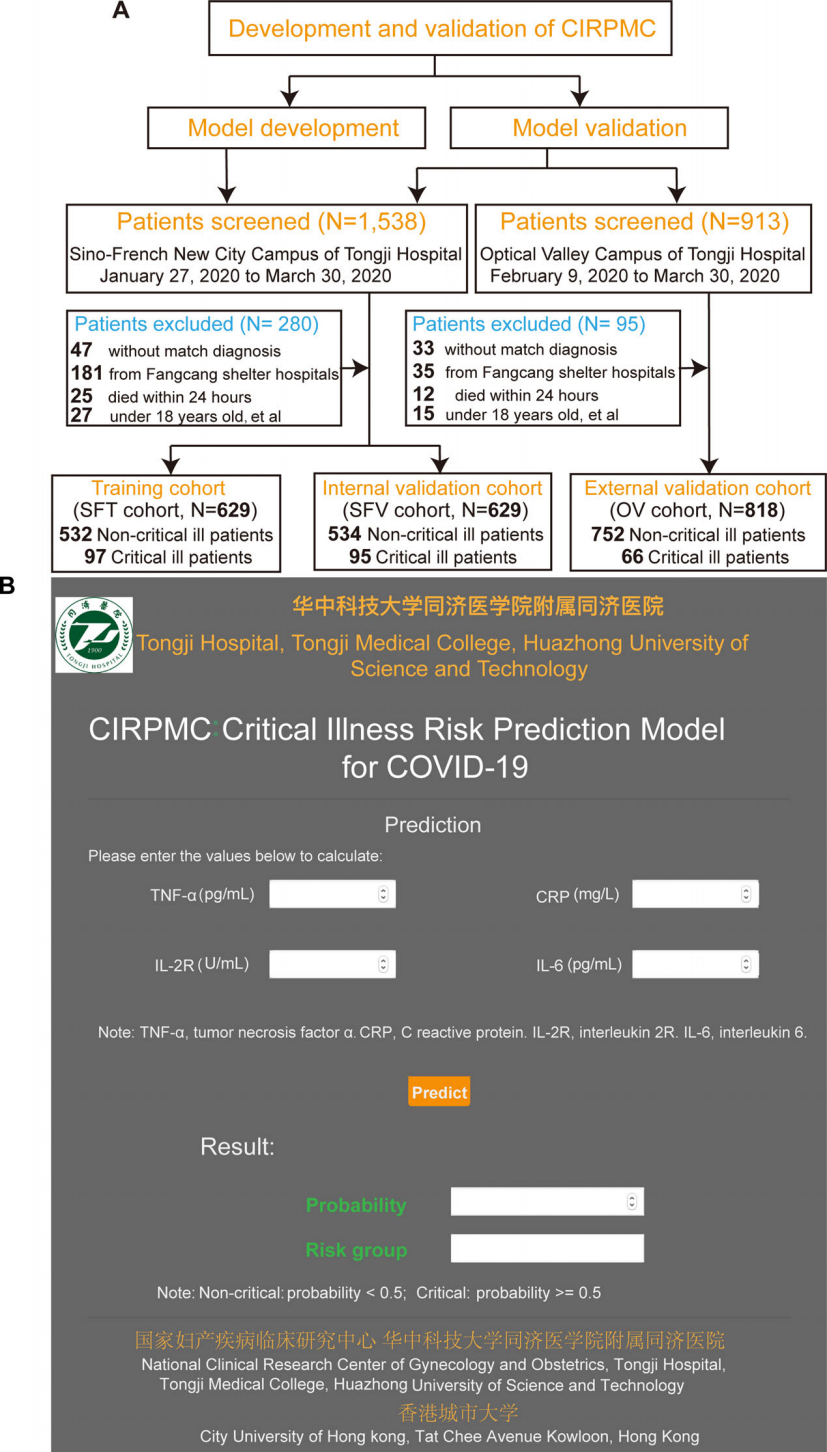
Working flow of the study. A, Study design. B, Illustration of the online prediction model‐CIRPMC. Abbreviations: CIRPMC, critical illness risk prediction model for COVID‐19; CRP, C reactive protein; IL‐2R, interleukin 2 receptor; IL‐6, interleukin 6; OV cohort, external validation cohort of Optical Valley Campus of Tongji Hospital; SFT cohort, training cohort of Sino‐French New City Campus of Tongji Hospital; SFV cohort, internal validation cohort of Sino‐French New City Campus of Tongji Hospital; TNF‐α, tumor necrosis factor α

Certain interpretability is a strength of CIRPMC. In accord with previous reports, we found that the expression levels of four contributive inflammatory cytokines (CRP, TNF‐α, IL‐2R, and IL‐6) were significantly higher in critically ill patients than those in noncritically ill patients.^5^ Another strengths of CIRPMC are its stability and generalizability. Four features used for prediction are readily accessible and frequently monitored in routine clinical practice. Besides, they are relatively objective, solid, and less susceptible to human memory bias, suggesting that CIRPMC is not susceptible to human interference and has strong generalization to be extended to other medical institutions.

During the pandemic, there has emerged many studies on prognosis prediction of COVID‐19.^6^, ^7^, ^8^ However, the sample size of most studies is small, thus harboring risks of overfitting.^6^, ^7^ Moreover, most studies lack independent external validation or the number of patients within external validation is limited,^9^, ^10^ which can impair the reproducibility and credibility of models. Our study is with larger sample size, independent external validation, detailed patient description, and relatively long observation time (18‐20 days).

However, the study has some limitations. First, patients included are primarily locals in Wuhan. Data from multiple provinces or countries could further improve the applicability and robustness of models. Besides, the prognostic implication of CIRPMC has not been evaluated in prospective cohorts due to the retrospective nature of this study.

In conclusion, this retrospective, multicenter study showed CIRPMC with readily available features holds great potential in accurately and timely (approximately 20 days in advance) identifying COVID‐19 patients prone to develop into critical illness. The model held strong stability, generalizability, universality, and wide prediction horizon to be easily extended to areas with limited medical resources. The proposed model potentially assists clinicians to locate the patients with a higher priority to be early intervened and intensively monitored, and eliminate delays to maximize the number of survivors during the rapidly developing global emergency. Equipped with high predictive performance, the online calculator CIRPMC deserves to be proceeded with. However, these findings warrant further validations in prospective clinical trials.

## CONFLICTS OF INTEREST

The authors have no conflicts of interest to declare.

## ETHICS APPROVAL AND CONSENT TO PARTICIPATE

This study was approved by the Research Ethics Commission of Tongji Hospital of Huazhong University of Science and Technology (TJ‐IRB20200406) in view of the retrospective nature of the study and all the procedures performed were part of the routine care. The trial has been registered in the Chinese Clinical Trial Registry (ChiCTR2000032161). The informed consents were waived by the Ethics Commission of Tongji Hospital of Huazhong University of Science and Technology.

## AUTHOR CONTRIBUTIONS

QG had full access to all data in the study, took responsibility for the integrity of data, and the accuracy of the data analysis. YG designed the study. LC did the analysis. YG, LC, and HL interpreted the data and wrote the paper. SZ, XF, YW, TJ, YY, JC, XJ, DL, XF, SW, RY, YY, SX, XX, PC, QM, XJ, and YW provided patients’ samples and clinical data, entered the data into database, and double‐checked the data. QG, SL, CL, and DM advised on the conception and design of the study. All authors vouched for the respective data and analysis, approved the final version, and agreed to publish the manuscript.

## Supporting information

Supplement Methods.
Figure S1: Visualization of the denosing and filtering process.
Figure S2: Visualization of the imputation process.
Figure S3: Statistical analysis of four features selected by Lasso.
Figure S4: Relative feature importance of SVM model.
Figure S5: Calibration curves of SVM model in cohorts.
Table S1: Baseline characteristics of individuals by cohorts.

## Data Availability

The data contain information that could compromise research participant privacy, and so are not publicly available. Data supporting the findings of this study are available from the corresponding author upon reasonable request.
