# Supplementary material for: CIRPMC: An online model with simplified inflammatory signature to predict the occurrence of critical illness in patients with COVID‐19
Source: Clin Transl Med. 2020 Oct 21;10(6):e210. doi: 10.1002/ctm2.210 (PMC7577323; doi:10.1002/ctm2.210)
Supplement: Supplementary file 1 — Supplement Methods. Figure S1: Visualization of the denosing and filtering process. Figure S2: Visualization of the imputation process. Figure S3: Statistical analysis of four features selected by Lasso. Figure S4: Relative feature importance of SVM model. Figure S5: Calibration curves of SVM model in cohorts. Table S1: Baseline characteristics of individuals by cohorts. [file CTM2-10-e210-s001.docx]

**Supplement Methods**

**Study design and participants**

2,451 consecutive discharged or dead COVID-19 patients were included from two designated hospitals (Sino-French New City Campus of Tongji Hospital, SF; Optical Valley Campus of Tongji Hospital, OV) for COVID-19 between January 27 and March 30. Among them, 80 patients were excluded from the diagnosis of COVID-19 according to the 7th edition of the Diagnosis and Treatment Protocol of COVID-19 by the National Health Commission. We further excluded 216 patients that were transferred from mobile cabin hospitals for the requirement of isolation, 37 patients that died within 24 hours of admission, and 42 patients under 18 years old, pregnant, re-hospitalization, or discharged for special reasons such as dialysis. Eventually, 2,076 patients were included for model training and validations.

We randomly partitioned 50% and 50% of participants from SF into training cohort (SFT cohort) and internal validation cohort (SFV cohort), respectively. Accordingly, participants from two hospitals were divided into three independent cohorts, including a training cohort (SFT), an internal validation cohort (SFV), and an external validation cohort (OV). There is no overlap across cohorts at any level. Critical illness was a composite endpoint defined as invasive ventilation, admission to the intensive care unit, or death according to previous studies on the severity of serious infectious diseases ^1,2^. Features consist of 15 inflammatory/immune markers [C reactive protein (CRP), procalcitonin (PCT), interferon-γ (IFN-γ), tumor necrosis factor α (TNF-α), interleukin 1β (IL-1β), interleukin 2 receptor (IL-2R), interleukin 4 (IL-4), interleukin 6 (IL-6), interleukin 8 (IL-8), interleukin 10 (IL-10), immunoglobulin A (IGA), immunoglobulin G (IGG), immunoglobulin M (IGM), SARS-COV-2 specific antibody IgM (C-IGM), SARS-COV-2 specific antibody IgG (C-IGG)], and outcome data were collected at admission from electronic health records (EHRs) using the same designed data collection form across cohorts. Trained researchers entered and double-checked the data independently.

**Data preprocessing**

The features from EHRs usually contain noisy and missing entries (Figure S1A). To address this issue, we first filtered out the features with data missing more than or equal to 20% across the cohorts. As a result, the number of features was reduced from fifteen to eight (Figure S1B). We then imputed the missing entries in two hospitals separately. missForest was utilized to estimate the missing value ^3^. Heatmap of the rest eight features before (Figure S2A, C) and after (Figure S2B, D) imputation was shown in Figure S2.

**Feature selection**

To identify the most predictive variables, we performed feature selection using Least Absolute Shrinkage and Selection Operator (LASSO) logistic regression ^1,4^. Lasso imposes the L1 penalty of features to the objective function, which forces the coefficients corresponding to those less predictive features to become zero. In this study, we considered features whose coefficients are zero as redundant features and removed them.

**Model development and evaluation**

In the present study, we fitted the features selected by Lasso into five machine learning models to predict the critical illness risk of patients with COVID-19. These models include logistic regression (LR), supported vector machine (SVM), gradient boosted decision tree (GBDT), k-nearest neighbour (KNN), and neural network (NN). As a binary classification problem, machine learning models output critical illness risk probability (*p*) ranged from 0 to 1 for each patient. We stratified patients with *p* < 0.5 as “low risk”, otherwise “high risk”. R library "caret" was utilized for model training and prediction with 10-fold cross-validation. The LR, SVM, GBDT, KNN, and NN were called with R packages "bayesglm", "svmLinear", “gbm”, “knn”, and "avNNet" with default settings, respectively. We normalized the data into mean=0 and SD=1 before training and prediction.

The predictive performance of models was evaluated by area under the receiver operating characteristics (AUC), accuracy, sensitivity, specificity, positive predictive value (PPV), negative predictive value (NPV), Cohen' s kappa coefficient (Kappa), F1 score, and Brier score.

**Statistical analysis**

All statistical analysis was performed with R (version 3.6.2). The receiver operating characteristics (ROC) curve and the area under the curve (AUC) analysis were conducted with R ''pROC' package. Accuracy (ACC), sensitivity (SE), specificity ^5^, positive predictive value (PPV), negative predictive value (NPV), Cohen' s kappa coefficient (Kappa), and F1 score were calculated with R "caret" and "epiR" packages. The calibration curve and Brier score were calculated with R package “rms”. Kaplan-Meier plot with log-rank test and univariate Cox analysis were conducted with R "survival" and "survminer" packages. Model importance was calculated using R package "caret". The correlation between selected features and critical illness status were calculated Spearman correlation. Mood’s median test (R package "coin") compares the medians of critical illness, and non-critical illness groups. P value less than 0.05 were considered significant.

**Supplementary references**

1. Liang W, Liang H, Ou L, et al. Development and Validation of a Clinical Risk Score to Predict the Occurrence of Critical Illness in Hospitalized Patients With COVID-19. *JAMA Intern Med.* 2020.

2. Gao HN, Lu HZ, Cao B, et al. Clinical findings in 111 cases of influenza A (H7N9) virus infection. *N Engl J Med.* 2013;368(24):2277-2285.

3. Stekhoven DJ, Bühlmann P. MissForest--non-parametric missing value imputation for mixed-type data. *Bioinformatics.* 2012.

4. Fu H, Zhu Y, Wang Y, et al. Identification and Validation of Stromal Immunotype Predict Survival and Benefit from Adjuvant Chemotherapy in Patients with Muscle-Invasive Bladder Cancer. *Clin Cancer Res.* 2018;24(13):3069-3078.

5. Wynants L, Van Calster B, Collins GS, et al. Prediction models for diagnosis and prognosis of covid-19 infection: systematic review and critical appraisal. *BMJ.* 2020.

6. Yue H, Yu Q, Liu C, et al. Machine learning-based CT radiomics method for predicting hospital stay in patients with pneumonia associated with SARS-CoV-2 infection: a multicenter study. *2020.* 2020;8(14):859.

**Supplementary figure legends**

**
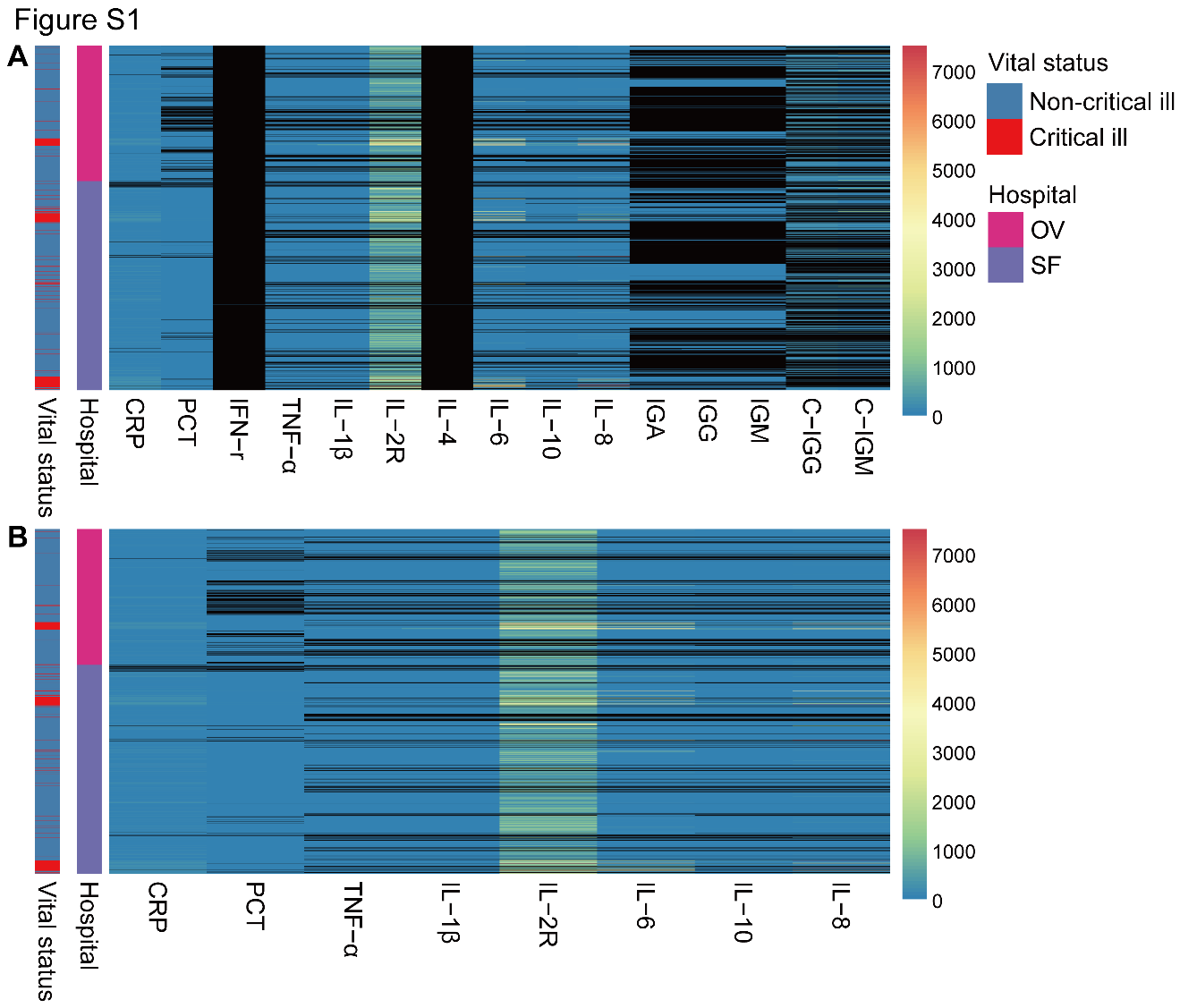
**

**Supplement figure 1: Visualization of the denosing and filtering process.**

**A** Heatmap of raw lab test data. **B** Heatmap of lab test data after removing lab test features with more than and equal to 20% missing entries across the SF and OV cohorts. Black tiles refer to missing entries.

*Abbreviations* CRP: C reactive protein; PCT: procalcitonin; IFN-γ: interferon-γ; TNF-α: tumor necrosis factor α; IL-1β: interleukin 1β; IL-2R: interleukin 2 receptor; IL-4: interleukin 4; IL-6: interleukin 6; IL-8: interleukin 8; IL-10: interleukin 10; IGA: immunoglobulin A; IGG: immunoglobulin G; IGM: immunoglobulin M; C-IGM: SARS-COV-2 specific antibody IgM; C-IGG: SARS-COV-2 specific antibody IgG; SF: Sino-French New City Campus of Tongji Hospital; OV: Optical Valley Campus of Tongji Hospital.

**
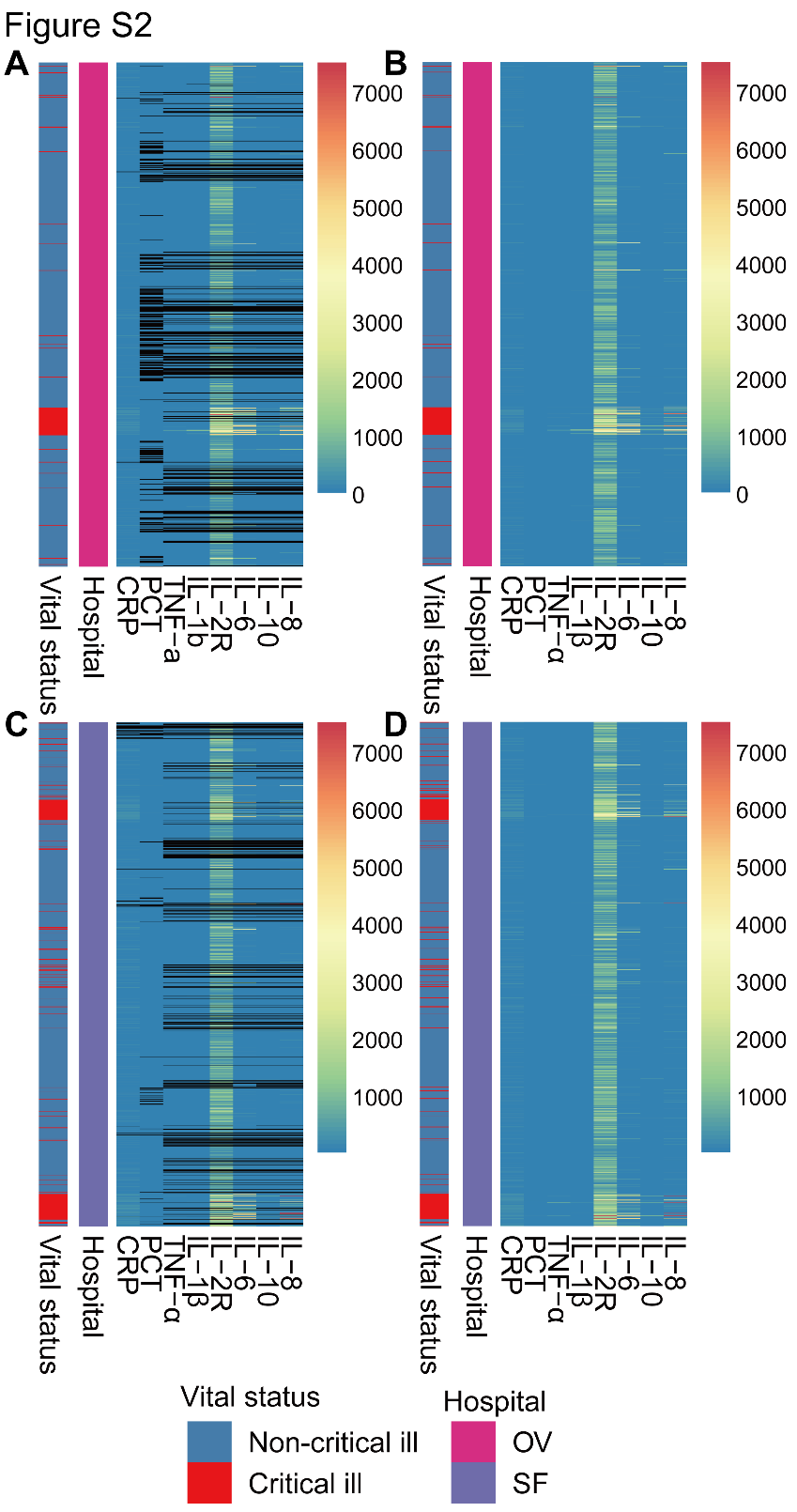
**

**Supplement figure 2: Visualization of the imputation process.**

**A, C** Heatmap of SF and OV lab test data before imputation. **B, D** Heatmap of SF and OV lab test data after imputation. Black tiles refer to missing entries.

*Abbreviations* CRP: C reactive protein; PCT: procalcitonin; TNF-α: tumor necrosis factor α; IL-1β: interleukin 1β. IL-2R: interleukin 2 receptor; IL-6: interleukin 6; IL-8: interleukin 8; IL-10: interleukin 10; SF: Sino-French New City Campus of Tongji Hospital; OV: Optical Valley Campus of Tongji Hospital.


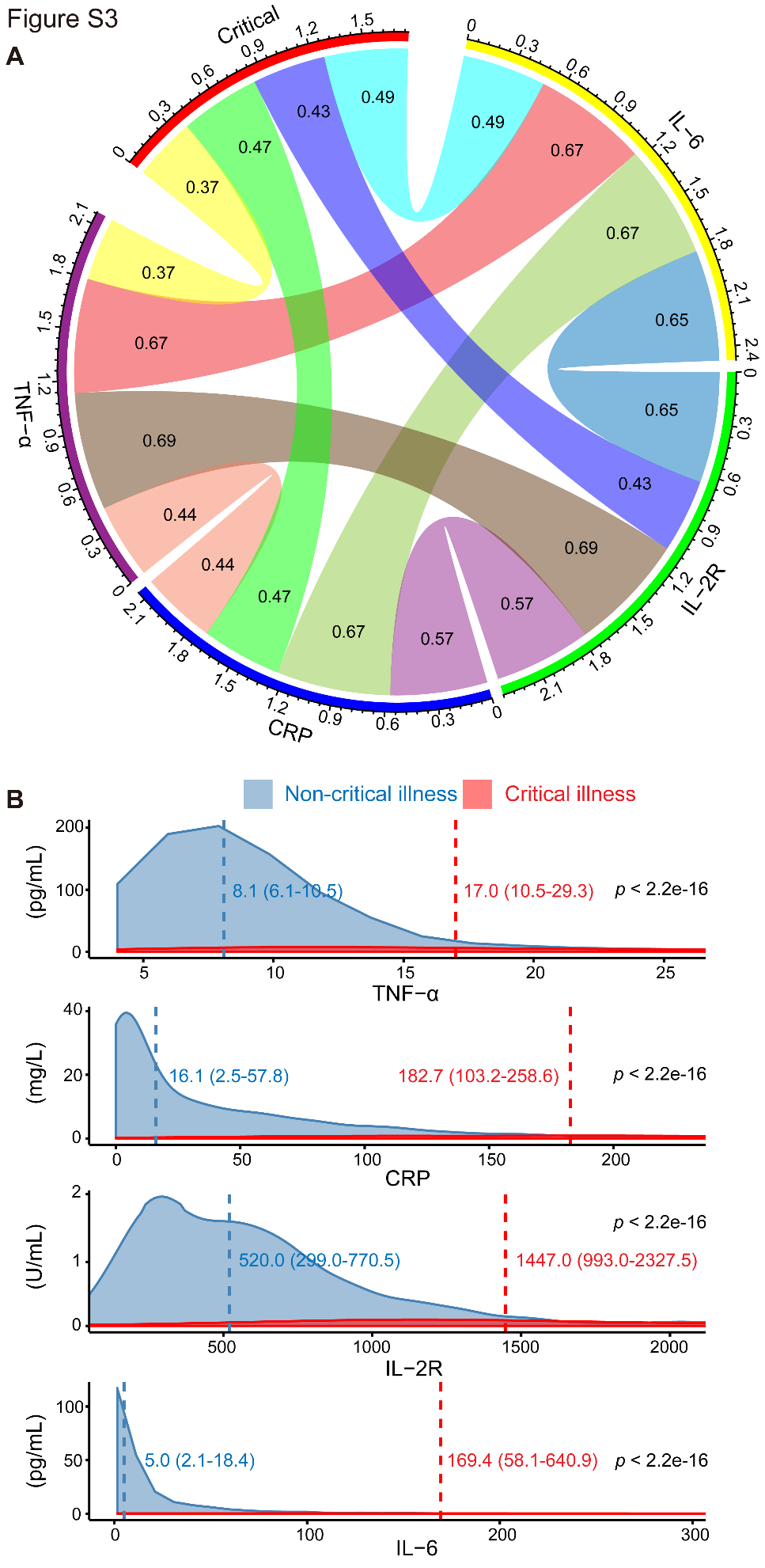


**Supplement figure 3: Statistical analysis of four features selected by Lasso.**

A, Spearman correlation of critical illness status and features. The wider chord, the stronger positive correlation is. B, Density plot of each feature across patients with different critical illness status, respectively. The vertical dashed line signifies the feature median value, interquartile range is also annotated. The significant test is Asymptotic Two-Sample Brown-Mood Median Test.

*Abbreviations* TNF-α: tumor necrosis factor α; CRP: C reactive protein; IL-2R: interleukin 2 receptor; IL-6, interleukin 6

**
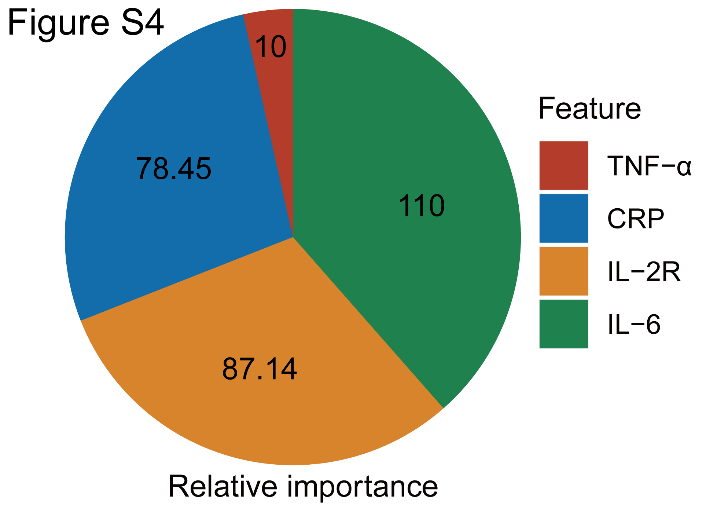
**

**Supplement figure 4: Relative feature importance of SVM model.**

*Abbreviations* SVM: supported vector machine; CRP: C reactive protein; TNF-α: tumor necrosis factor α; IL-2R: interleukin 2 receptor; IL-6: interleukin 6.


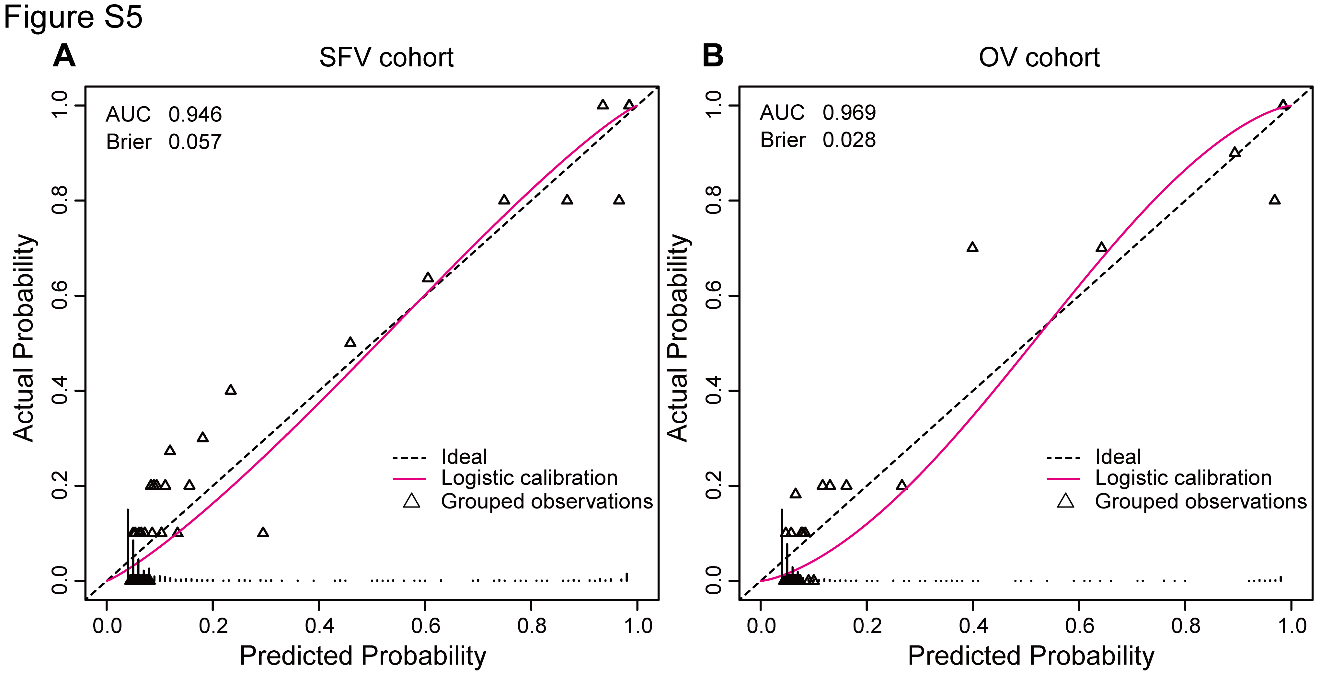


**Supplement figure 5: Calibration curves of SVM model in cohorts.**

Calibration curves of SVM model in **A** internal validation cohort SFV and **B** external validation cohort OV, respectively. The triangle represents the observation group. Each group contained an average of 20 observations. The dashed line is the ideal calibration curve. The bottom vertical lines refer to the predicted probability distribution. Red curve is the fitted linear logistic calibration curve.

*Abbreviations* SFV cohort: internal validation cohort of Sino-French New City Campus of Tongji Hospital; OV cohort: external validation cohort of Optical Valley Campus of Tongji Hospital; SVM: support vector machine.

**Supplement table S1: Baseline characteristics of individuals by cohorts.**

**Table S1. Baseline characteristics of individuals by cohorts.**

| **Characteristics** | **SFT cohort**  **Training set** | **SFV cohort**  **Internal Validation Set** | **OV cohort**  **External Validation Set** |
| --- | --- | --- | --- |
|  | **(N=629)** | **(N=629)** | **(N=818)** |
| **Demographics**  Age, years | 62 (52-70) | 62 (50-70) | 63 (50-70) |
| Sex |  |  |  |
| Female | 309 (49.13%) | 316 (50.24%) | 439 (53.67%) |
| Male | 320 (50.87%) | 313 (49.76%) | 379 (46.33%) |
| **Clinical characteristics** |  |  |  |
| Comorbidity number | 1 (0-2) | 1 (0-2) | 1 (0-2) |
| Comorbidity |  |  |  |
| Hypertension | 252 (40.06%) | 243 (38.63%) | 325 (39.73%) |
| Diabetes | 103 (16.38%) | 120 (19.08%) | 122 (14.91%) |
| CHD | 56 (8.90%) | 76 (12.08%) | 70 (8.56%) |
| CLD | 19 (3.02%) | 28 (4.45%) | 33 (4.03%) |
| Tumor | 29 (4.61%) | 29 (4.61%) | 37 (4.52%) |
| CKD | 10 (1.59%) | 11 (1.75%) | 11 (1.34%) |
| **Symptoms at admission** |  |  |  |
| Fever | 538 (85.53%) | 533 (84.74%) | 596 (72.86%) |
| Temp (max) ≥ 39℃ | 183 (29.09%) | 180 (28.62%) | 158 (19.32%) |
| Cough | 447 (71.07%) | 446 (70.91%) | 613 (74.94%) |
| Dyspnea | 300 (47.69%) | 302 (48.01%) | 284 (34.72%) |
| Sputum | 237 (37.68%) | 230 (36.57%) | 353 (43.15%) |
| Fatigue | 243 (38.63%) | 252 (40.06%) | 252 (30.81 %) |
| Diarrhea | 174 (27.66%) | 182 (28.93%) | 139 (16.99%) |
| Myalgia | 133 (21.14%) | 149 (23.69%) | 130 (15.89%) |
| Vomiting | 29 (4.61%) | 32 (5.09%) | 33 (4.03%) |
| **Vital status** |  |  |  |
| Critical illness | 97 (15.42%) | 95 (15.10%) | 66 (8.07%) |
| Non-critical illness | 532 (84.58 %) | 534 (84.90%) | 752 (91.93%) |
| **Follow-up,** days | 19 (10-27) | 20 (11-28) | 18 (12-24) |
|  |  |  |  |

Continuous variables were presented as median (interquartile ranges [IQR]) while categorical variables as counts and percentages ^6^. SFT, training cohort of Sino-French New City Campus of Tongji Hospital. SFV, internal validation cohort of Sino-French New City Campus of Tongji Hospital. OV, Optical Valley Campus of Tongji Hospital. CHD, Coronary heart disease. CLD, Chronic liver disease; CKD, Chronic kidney disease.

*Abbreviations* SFT cohort: training cohort of Sino-French New City Campus of Tongji Hospital; SFV cohort: internal validation cohort of Sino-French New City Campus of Tongji Hospital; OV cohort: external validation cohort of Optical Valley Campus of Tongji Hospital; CHD: coronary heart disease; CLD: chronic liver disease; CKD: chronic kidney disease.
